# Investigation of CMOS Multiplexer Jet Matrix Addressing and Micro-Droplets within a Printhead Chip

**DOI:** 10.3390/mi8120346

**Published:** 2017-11-29

**Authors:** Jian-Chiun Liou, Cheng-Fu Yang

**Affiliations:** 1 School of Biomedical Engineering, Taipei Medical University, Taipei 11031, Taiwan; jcliou@tmu.edu.tw; 2 Department of Chemical and Materials Engineering, National University of Kaohsiung, Kaohsiung 811, Taiwan

**Keywords:** complementary metal-oxide-semiconductor (CMOS) multiplexer, jets matrix, micro-droplet, printhead chip

## Abstract

In this study, we demonstrate and investigate a new droplet injection design. We create a thermal inkjet (TIJ) printhead using an application-specific integrated circuit system and bulk micromachining technology (microelectromechanical systems). We design inkjet printhead chips with a new structure and investigate their properties. For the new structure, the integration of complementary metal-oxide-semiconductors (MOSs) and enhancement-mode devices, as well as power switches and a TIJ heater transducer, enables logic functions to be executed on-chip. This capability is used in the proposed design to address individual jets with even fewer input lines than in matrix addressing. A high number of jets (at least 896) can be addressed with only 11 input lines. E1 (Enable 1) and E2 (Enable 2) are set up dependently, and they have the ability to reverse their signals in relation to each other (i.e., if E1 is disabled, E2 is enabled and vice versa). The E1 and E2 signals each service 448 jets. If one of the MOSs is turned on, then it corresponds to a power line with a similar function. If an addressing gate terminal of the other MOS has a discharge action, then we can control a different heater to generate heating bubbles in the jet inks. The operating frequency for addressing these measurements is 18 kHz in normal mode, 26 kHz in draft mode, and 16 kHz in best mode.

## 1. Introduction

Conventionally, inkjet printers generally use electro-thermal bubble-jet technology, in which an electrical pulse heats a small ink tank until bubbles in the ink are squeezed out. The shortcoming of this method is that the rapid heating, expansion, and compression of the ink expel it onto the printing paper, forming unwanted dots on the printing objects. Inkjet technology increases the stability of the droplet color to achieve both high-speed and high-quality printing [1,2,3,4,5,6], in which the ink droplets have uniform size and shape, and the consistency of the ink concentration enhances the image quality. However, at high temperatures, it is difficult to control the direction and shape of the ink droplets [7,8,9,10,11,12,13], and high-precision control of the droplets is crucial to achieve high-quality printed products. Thermal inkjet (TIJ) printing forces the ink into a tiny capillary. The system rapidly heats the ink to boiling point with a miniature heating pad, generating a minute vapor bubble that expands a drop of ink into the top of the capillary. The heating then stops and the ink cools, causing the vapor to condense and contract, so that the flow of ink ceases until steam next generates an ink droplet [14,15,16,17]. The speed of inkjet printing is a key technical indicator in fabricating a high-quality printer. To date, increasing the inkjet ejection frequency requires an increase in the number of holes and heaters on a single printhead.

Printing ink that is made of a variety of substances can be sprayed on different substrates. The ink drops are printed in tiny, picoliter units, and precision at the micrometer (μm) level that is relative to the position of the unit is required to achieve accurate printing. A specialized precision printing method operates at the microscopic level to produce flexible printed electronics, photovoltaics, color filters via screen printing, and sequences of genetic material. Diverse applications of inkjet technology have been pioneered in a wide range of industrial fields [18,19,20,21,22].

Recently, many electronic products and processes have matured, including organic crystals, organic memory devices, three-dimensional electronic components, embedded passive components, system packaging, film flip chip packaging, color filter manufacturing, film alignment processes, liquid crystal injection, and electrodes. For a variety of printable electronic components and related maskless printing technology, international manufacturers are actively involved in developing inkjet materials, processing technology, and planting platform. For high-end inkjet applications, the trend is to fine-tune the base of the inkjet drive circuit in pursuit of precision. This includes developing ink droplet nozzle technology and uniform ink droplet printing technology.

The design that is proposed in this study can drive numerous inkjet head designs on jet printing systems. The less the variation in the ink droplets that are sprayed from the holes on each nozzle, the better the printing quality. Hence, one of the research and development targets in this field is the uniform printing of ink droplets. This study proposes a new design for independent nozzle drive technology equipped with an inkjet method for a fluid control technology platform. The technique is specific to the area of the substrate, where the material attaches to form a picture pixel by pixel. This technology can work without a mask or high-level application process, reforming the existing traditional process and leading to new industrial applications, thereby enhancing the value of inkjet technology for industrial markets.

The current problem is the need for high-resolution displays for emerging manufacturing applications. The use of inkjet-derived technology is a promising new area, but to be practical, a stable production process with high reliability is necessary. This paper presents a thermal bubble-jet inkjet printhead wafer system, which can be implemented using platform-coating technology. In this system, one ink nozzle contains several holes that are arranged in a single, tightly packed line. Each of the holes has a separate dedicated power actuator that can be controlled by signals from the jet printing control system to determine how many holes in the nozzle should perform the printing action. This mechanism is called drop-on-demand.

For ink to be ejected from any of the holes, a driving waveform must generate an ink droplet using the thermal resistance of the bubble film pressure through the injection hole. This enables the inkjet head production process to support more industrial applications. Inkjet head manufacturers are developing small-volume ink droplets. To mitigate problems with fluid flow rate, film thermal resistance, and compatibility, it is necessary to make the holes on each nozzle jet produce precise, accurate, and consistent ink drops. In inkjet head manufacturing, each nozzle must meet these stringent requirements.

## 2. Design of the Multiplexer Inkjet Chip System

### 2.1. The Logic Processing Circuit

**Figure 1:**
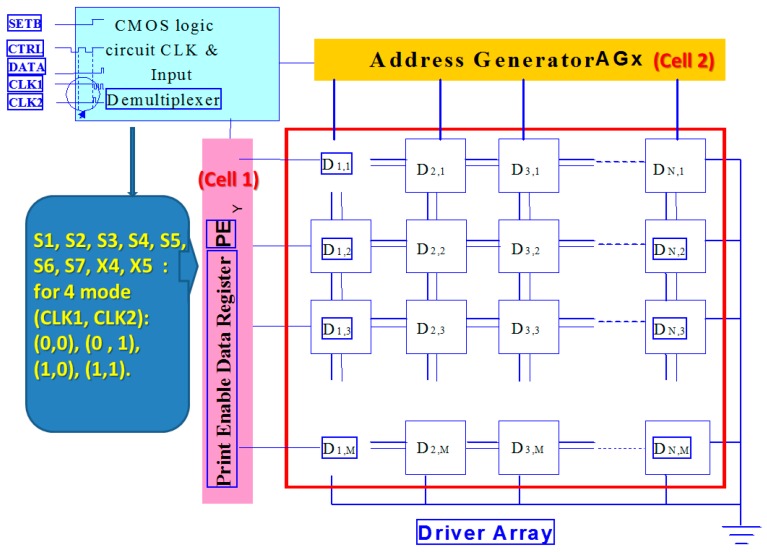
Multiplexer ink jet head of the uppermost layer within the inkjet driver wafer.

**Figure 2:**
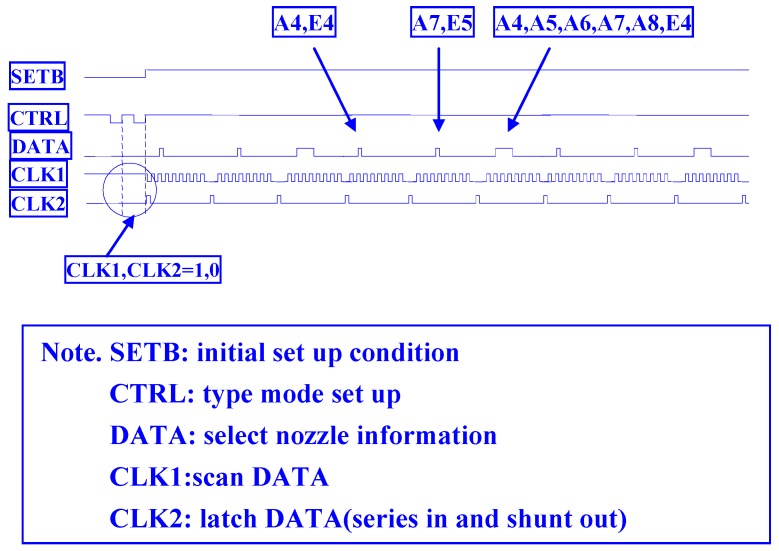
Input signal of the inkjet driver chip.

For the multiplexer inkjet head driver circuit, the most important function is to input the five signals for logic operation to the microelectromechanical element side, which specifies a group of nozzle hole operations. These are not geometrically adjacent; therefore, they do not affect each other. Figure 1 shows the upper layer of the multiplexer inkjet head within the inkjet driver wafer. The logic signal processing unit must determine whether the DATA signal is to be either positive or negative. The chip signal for CELL2 is the last count signal, CL1 in addition to CL2 and CLK, where CL2 is the positive edge of the trigger, CL1 is the negative edge of the trigger, and CLK is the general data in place after the latch is down to the output. The signals S1, S2, S3, S4, S5, S6, S7, X4, and X5 are sent by CLK1, CLK2, CTRL, SETB, and the DATA external logic input to detect varying signal results. When SETB is “0” and DATA is “1”, CTRL sends two pulses to scan the combination of CLK1 and CLK2 at that moment where (CLK1, CLK2) is (0, 0), (0, 1), (1, 0), or (1, 1). Figure 2 shows the input signal of the inkjet driver chip, which includes a first-level logic processing unit. CELL1 is the unit for the enable signal, and CELL2 is an addressing unit combining the CELL1 and CELL2 logic operation units, as well as the voltage adjustment output unit. The logic processing circuit for controlling the array element of large microelectromechanical systems (MEMS) is as follows. The inkjet drive wafer is determined by the inkjet speed and the resolution of the inkjet system. To achieve high speed and high resolution, the inkjet drive circuit is designed so that an enable signal can cover multiple addressing nozzles. It does not perform the logic operation before the current is switched, although a cycle can specify the maximum number of holes. Another approach is to increase the number of enable cycles to control the huge nozzle array.

**Figure 3:**
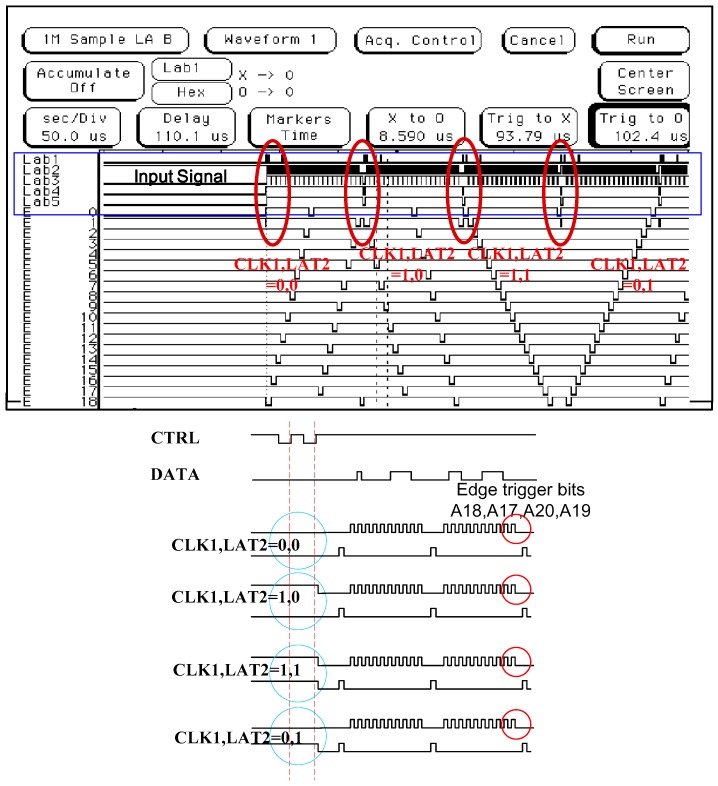
Entire 16 × 28 thermal resistance thin film control driver circuit signal and field programmable gate array verification result.

Figure 3 shows the entire 16 × 28 thermal resistance thin film control driver circuit signal and the field programmable gate array (FPGA) verification result. The first type ((CLK1, LAT2) is (0, 0)) drives each micro-heater addressing from high to low latches (E28, E26, …, E27, E25, …, E1, E3, …, E27, E2, E4, …, E28), and the second type ((CLK1, LAT2) is (1, 0)) drives each micro-heater addressing from a low count to a high latch. The two designs in the micro-heater components will cause thermal resistance to produce thermal interference. The geometry of the thermal resistance array has been fixed. The use of an inkjet timer to achieve driving operation can ensure multiple circuit control, so thermal interference can be avoided.

**Figure 4:**
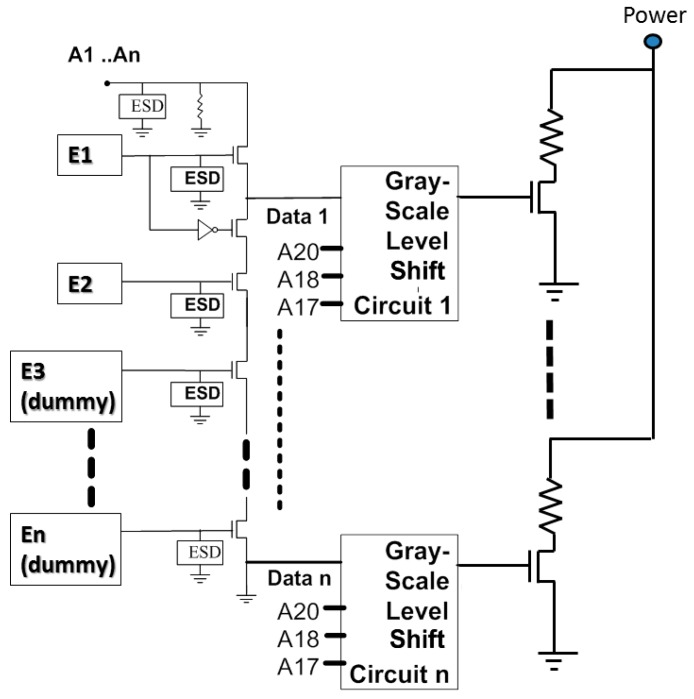
Driver array cell.

The third type ((CLK1, LAT2) is (1, 1)) drives each thermal resistance addressing from low to high latches (E0, E1, …, E28). The fourth type ((CLK1, LAT2) is (0, 1)) drives each thermal resistance addressing from a high count to a low latch (E28, E27, …, E0). The other two designs allow for a wide range of thermal resistance between the components, but they must also be operated one after the other. A serial CLK1 scanning signal latches 20 bits at a time. The last four bits (A18, A17, A20, A19) are used in modulating the amplitude of the output power. Voltages V_0_, V_1_, V_2_, V_3_, V_4_, V_5_, V_6_, and V_7_ are selected to be related to the thermal resistance switch module by three to eight decoders, based on the displacement harmonics wavelength signal. Figure 4 shows the driver array cell. The cell is the final stage of the inkjet chip with a dummy terminal, which can be extended to drive more jets in the future. This novel control technology can be extended to drive more nozzles, which can be increased from 16 × 28 thermal resistance to 16 × 56 thermal resistance. In other words, we can increase the number of latches to achieve this novelty function—for example, E56, E54, ..., E28, E26, ..., E4, E2, ....

**Figure 5:**
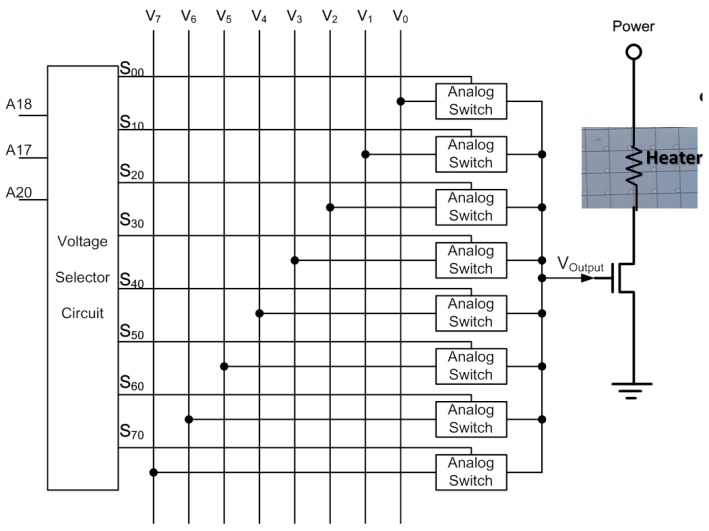
Power grayscale output cell.

The power grayscale output signals are supplied as the thermal resistance elements of the inkjet wafer system (Figure 5). This crucial unit comprises a switch circuit and a voltage selection circuit, wherein the switch is composed of multiple analog switches. This crucial unit consists of a P-channel metal-oxide-semiconductor (PMOS) and an N-channel MOS (NMOS), and is connected in parallel to the voltage selection circuit. The voltage selection circuit outputs eight select signals (S_00_ to S_70_) of from one to eight decoder circuits, which connect to the source side of the PMOS. The inverted signals (S_00_ to S_70_) are sent to the source side of the NMOS. The gate terminals of the two halogens are commonly connected to one of voltage outputs within the range V_0_–V_7_. The drain terminals of both MOSs are connected to V_Output_ to supply microthermal resistance.

A multiprocessing signal circuit can change the frequency and output amplitude based on the size of the functional circuit system. Multiprocessing signal functionality lies in the selection of a large array of thermal resistance switches (addressing). Because of this, these signals can quickly point out which thermal resistance to activate, and then perform ink-jetting after selection.

### 2.2. The MEMS Processes

**Figure 6:**
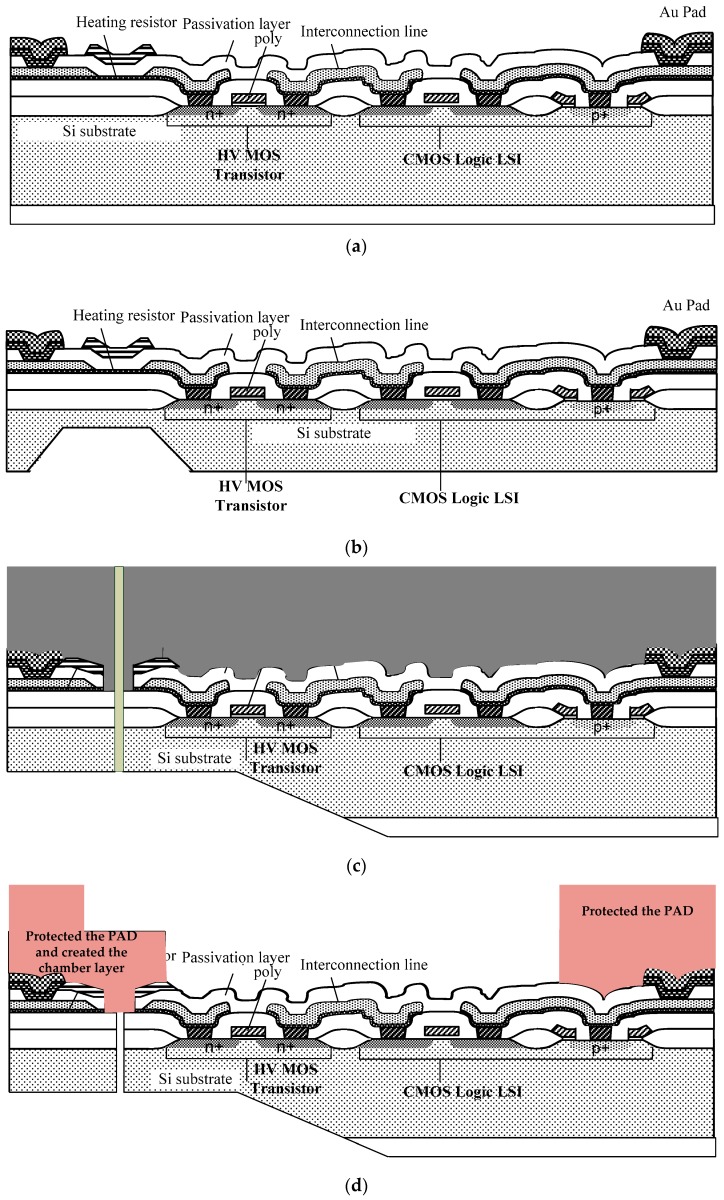

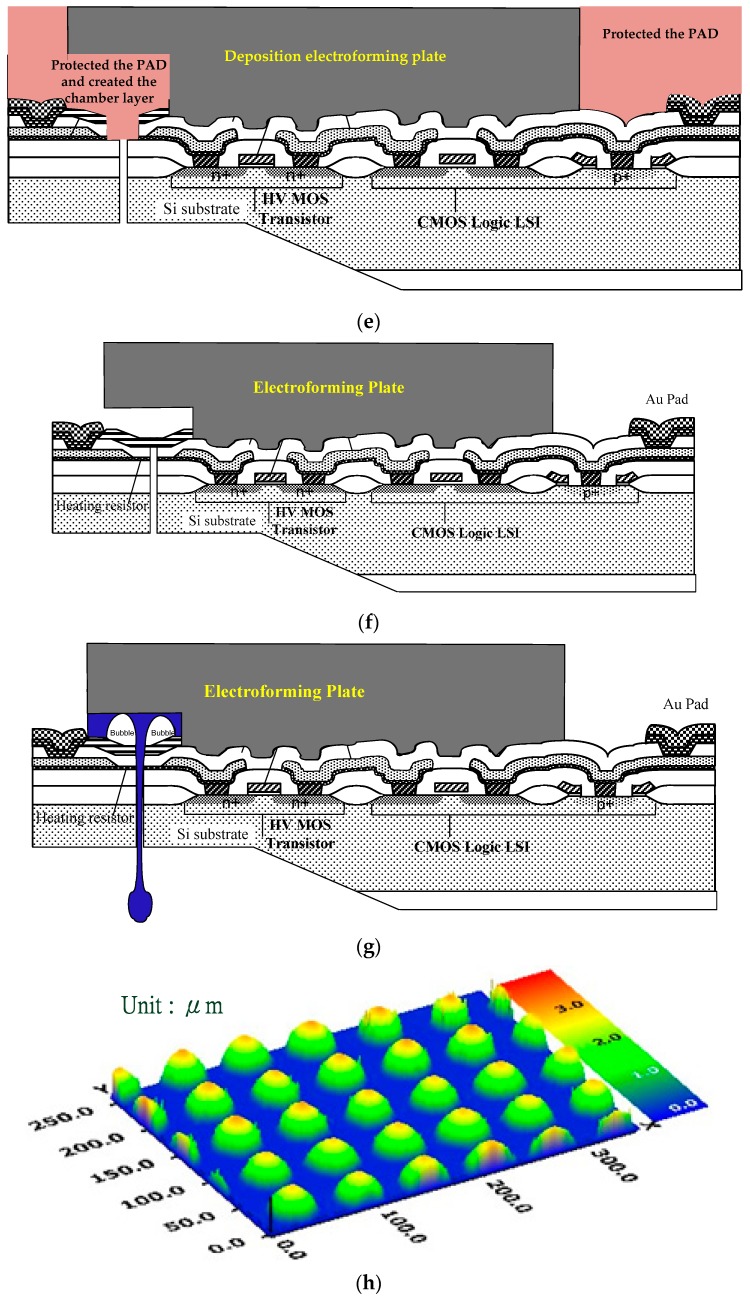
(**a**) Complementary metal-oxide-semiconductor (CMOS) circuits substract; (**b**) Partially thin substrate orifice area as a nozzle plate; (**c**) CMOS circuit by covering the photoresistance on the 3D sacrificial layer for perforation; (**d**) The protected the PAD and created the chamber layer; (**e**) Deposition electroforming plate; (**f**) Removed the protected the PAD and created the chamber layer and finished a liquid MEMS ink-jet head; (**g**) Thermal bubble generation droplet; and, (**h**) droplet arrangement profile.

For the design of the MEMS system, the micromachining printhead nozzle is contained in a liquid chamber and the nozzle is constructed and integrated as a monolithic chip. The flow of MEMS part processes are fabricated directly on top of complementary metal-oxide-semiconductor (CMOS) wafer. The detailed process is described in Figure 6 below. 

In the integration of the circuit and the MEMS, the investigated issues can be integrated as a monolithic chip. After ICP vertical etching on the Si substrate by MEMS processes to form nozzles, the driving singles stil need l presenting correct driving waveform and high driver performance by CMOS circuits, as Figure 6a shows. The next step is to partially make the substrate orifice area thin as as a nozzle plate, as shown in Figure 6b. The solve method of these problems is to achieve the best protected CMOS circuit by covering the photo-resistance on the three-dimensional (3D) sacrificial layer for perforation, as Figure 6c shows. The protected connection terminals of input and output signals (PAD) and created the chamber layer is shown in Figure 6d. Figure 6e shows the deposition the electroforming plate layer and Figure 6f shows the removed the protected the PAD and created the chamber layer and finished a liquid MEMS ink-jet head. Figure 6g shows the thermal bubble generation droplet. The droplet arrangement profile is shown in Figure 6h.

Using ICP vertical etching on the Si substrate by MEMS processes to form nozzles can create smaller nozzle sizes than other methods [23,24]. The assembled in nozzles and CMOS circuits of Timo Lindemann’s research, the printheads are made by a combination of a standard printhead CMOS substrate with a three-dimensional (3-D) structured polyimide nozzle plate, and that can avoid the results of three-layer assembly. The nozzle plate is assembled with the substrate using a adhesive layer with an alignment accuracy of 5 μm. The integrated nozzle plate leads to a control of the printhead geometry and saves one processing step in production [23]. The assembled in nozzles and CMOS circuits of Regan Nayve’s research, the printhead has been fabricated by dicing the bonded wafer, which consists of a bubble generating heater plate (CMOS circuits) and a Si channel plate. The Si channel plate consists of ink inlet and ink chamber formed by KOH etching and nozzles that are formed by RIE process. The ink inlet is a through-hole structure to supply ink from the ink tank to the ink chamber. The ink chamber is connected to each nozzle [24].

In this study, the ICP vertical etching of a bulk nozzle structure can achieve a spray cycle time of 40 μs. The nozzle spacing is getting smaller and smaller, that will enhance the printing resolution and printing speed. Other printheads cannot achieve this spray cycle time without ICP vertical etching of the bulk nozzle structure.

## 3. Experiment and Signal Measurement

**Figure 7:**
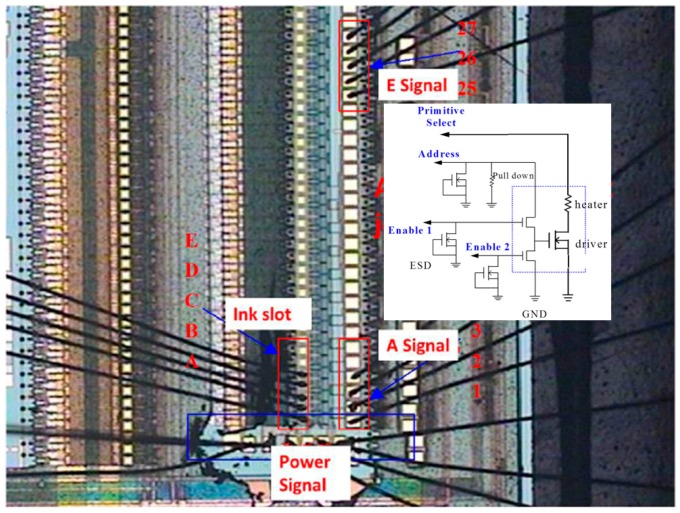
Photo of the chip and schematic diagram of the drive circuit.

The total inkjet chip system for the smart printhead is shown in Figure 7. It is based on a 2.5 μm NMOS process and follows the physical design rules of a 0.25 μm two-layer material of poly and four-layer material of metal, a 5 V supply for the gate terminal, and a 24 V supply for the drain terminal (2P4M 5 V/24 V). High voltage forms a symmetric high-voltage device with lower turned on resistor (Ron) and higher current driving. It has a symmetric HV device structure between the source and drain terminals.

**Figure 8:**
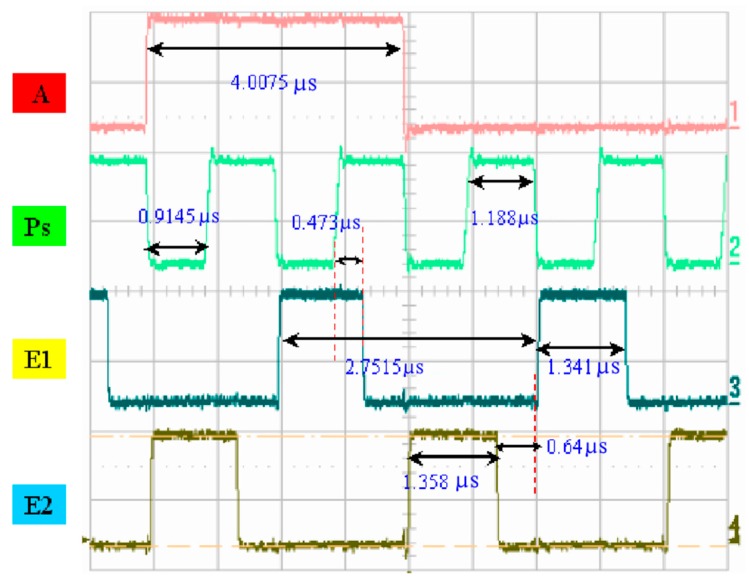
Time-scale testing.

**Figure 9:**
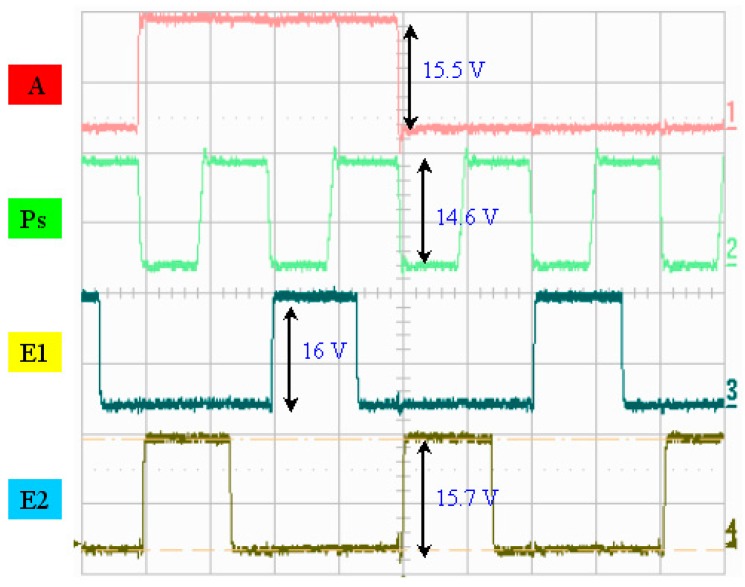
Voltage-scale testing.

**Figure 10:**
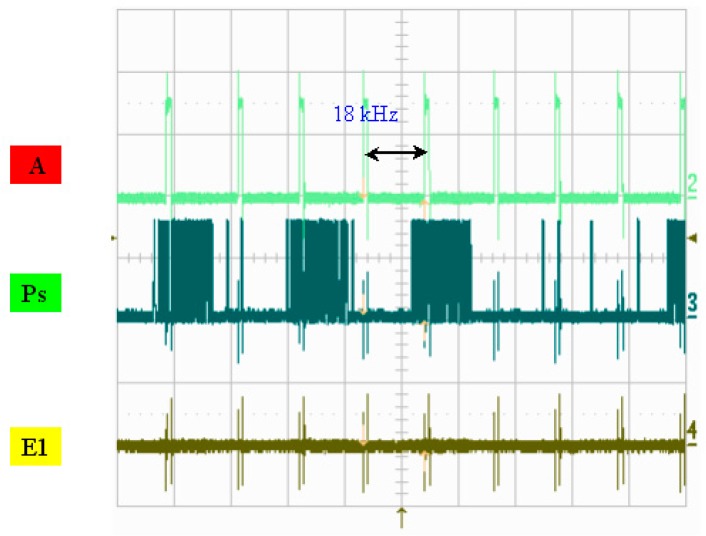
Frequency testing: normal mode.

**Figure 11:**
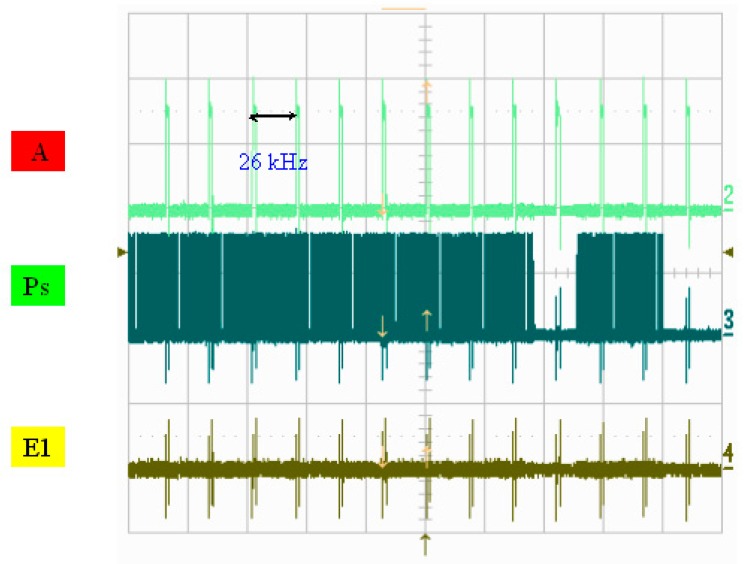
Frequency testing: draft Mode.

**Figure 12:**
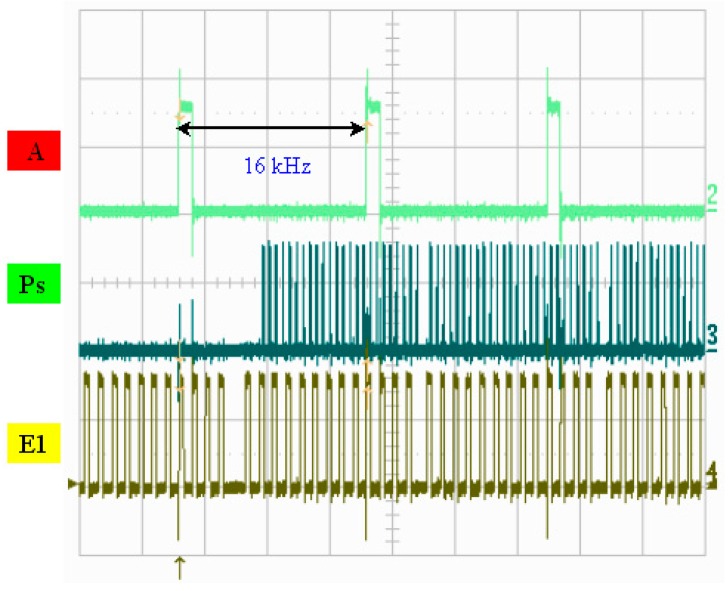
Frequency testing: best mode.

A photo of the chip and a schematic diagram of the drive circuit are presented in Figure 7. Figure 8 illustrates the measured width of each pulse signal and their mutual correspondence, demonstrating that the width of pulse A was 4 μs and that the width of pulse P was 1.18 μs, where E1 and E2 were determined to be 1.34 μs and 1.35 μs, respectively. E1 and E2 can be set up so that they have the reverse signal to each other. If E1’s signal is disabled, E2 is enabled, and vice versa. If one of the MOSs is turned on, corresponding to the same P and A of the other MOS’s discharge action, then a different heater will be controlled. In addition, P, A, and E1 are simultaneously at a high level. That is, the heater actually only heats the bubble for 0.473 μs. Figure 9 shows the operating voltage of each signal: the signal of pulse A was 15.5 V, the signal of pulse P was 14.6 V, and the signals of pulses E1 and E2 were 16 and 15.7 V, respectively. Figure 10 shows frequency testing in normal mode, Figure 11 shows frequency testing in draft mode, and Figure 12 shows frequency testing in best mode. The respective operating frequencies for each mode were 18, 26, and 16 kHz. The two important novelties in the architecture of the control circuit are described below.

(1) This mode (E1 (Enable 1) and E2 (Enable 2)):

The important novelty in the investigated architecture is that the drive control circuit has the major advantage of reducing the mutual interference of the beads after the liquid droplets are ejected from the cavity. When the E1 signal (Enable 1) is set at “1”, it enables the E2 signal (Enable 2) to be changed to “0”. This control technology can be extended to other similar signals—for example: (E1 (Enable 1), E2 (Enable 2), (E3 (Enable 3), E4 (Enable 4), E5 (Enable 5), E6 (Enable 6)) ... En (Enable n).

(2) The three signals “A”, “Ps”, and “E” pulse:

For the architecture of the investigated control circuit, the three signals must be at a high voltage level at the same time, then the corresponding switch of the heater will be turned on. The most innovative technology in this drive mode is that we can adjust the final output of the heater and control the size of the output liquid droplets. As shown in Figure 8 and Figure 9, the controlled heater has three signals that drive the “A”, “Ps”, and “E” pulses in the high voltage level. At the same time, these three signals dominate the power output of the heater. As shown in Figure 8, when the “A” signal selects the nozzle heater, the “Ps” and “E1” signals are also at high levels, with an overlapping time of 0.473 μs.

## 4. Results

**Figure 13:**
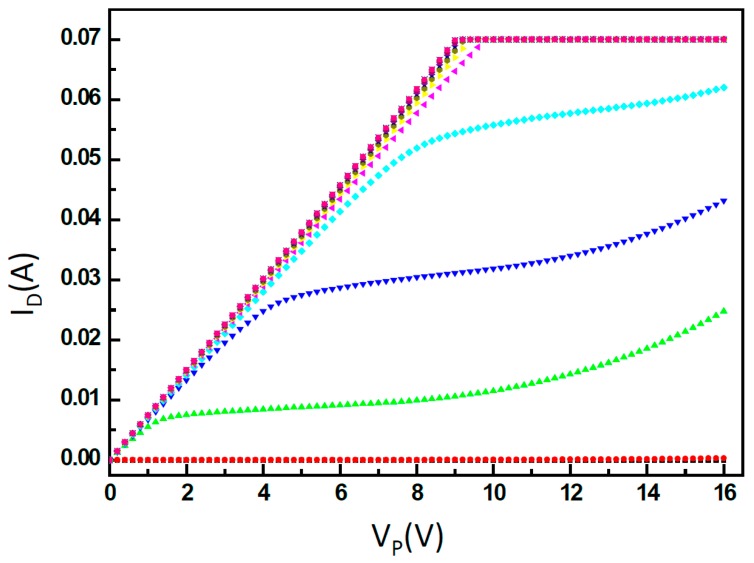
Current of drain versus the voltage of power (I_D_-V_P_) of the metal-oxide-semiconductors (MOS) driver’s electrical signal (driver with heater).

Chip design is a key component of the driver. The MOS driver must take a three-terminal electrical input. One terminal is the supply voltage V_G_, the second terminal is the PS side, and the third is the ground (GND) terminal, which measures the current I_D_. It also requires a 16 V DC bias on the E1 (or E2) input. The other En inputs are connected to ground with the G pad. The PS side of the inkjet chip is connected in series with the MOSFET driver. Therefore, if the I_D_-V_D_ diagram is measured for different V_G_ values, the MOSFET I_D_-V_D_ diagram of a long linear region is derived. The operating points of the MOS driver of the inkjet head were V_P_ = 14.6 V and V_GS_ = 15.5 V. Thus, the electrical signal of the MOS driver could be measured on the ink printhead. The signal line of the HP4155A semiconductor parameter analyzer was connected; the HP4155A input signal could then measure the MOS driver’s electrical signal. The results are shown in Figure 13. In addition, HP4155A was used to measure a direct current, which was different from the impulse signal that was driven by the printer, and under this condition, the current was in the range of 75–80 mA. If the thermal resistance on the inkjet wafer were continuously subjected to this large load current, then the thermal resistance of the film would suffer damage. Therefore, the current limit was set at 70 mA to protect the film’s thermal resistance during measurement.

**Figure 14:**
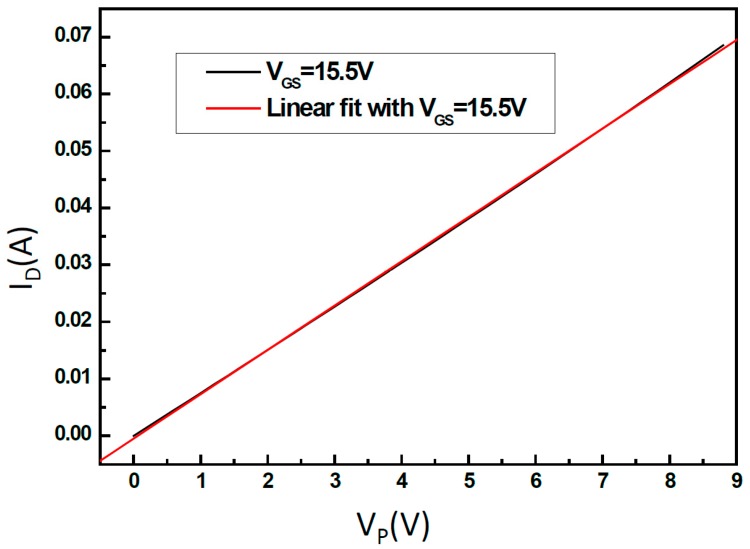
The I_D_-V_P_ linear curve of the inkjet chip (driver with heater).

The linear equation for this regression can be thought of as the I_D_-V_P_ curve operating in the linear region, as shown in Figure 14. Accordingly, one can infer that this line extends to an operating voltage for V_P_ = 14.6 V, and the current value is the actual drive current. This can be calculated by substituting V_P_ = 14.6 V into the curve. From the result in Figure 14, I_D_ = 113.6 mA can be derived and the operating point of R_total_ = (V_P_/I_D_) = (14.6 V/113.6 mA) ≈ 128.5 Ω can be obtained.

**Figure 15:**
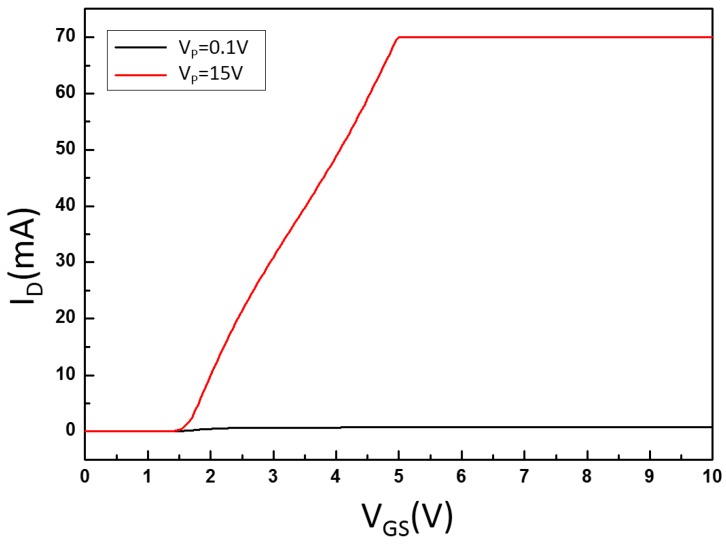
Threshold voltage of MOS driver.

In the measurement of the electrical signal of the HP4155A MOS driver, the starting voltage value of this MOS driver could be determined concurrently. The measurement results are presented in Figure 15. These show that the starting voltage of this MOS driver was V_GS_ = 1.5 V.

**Figure 16:**
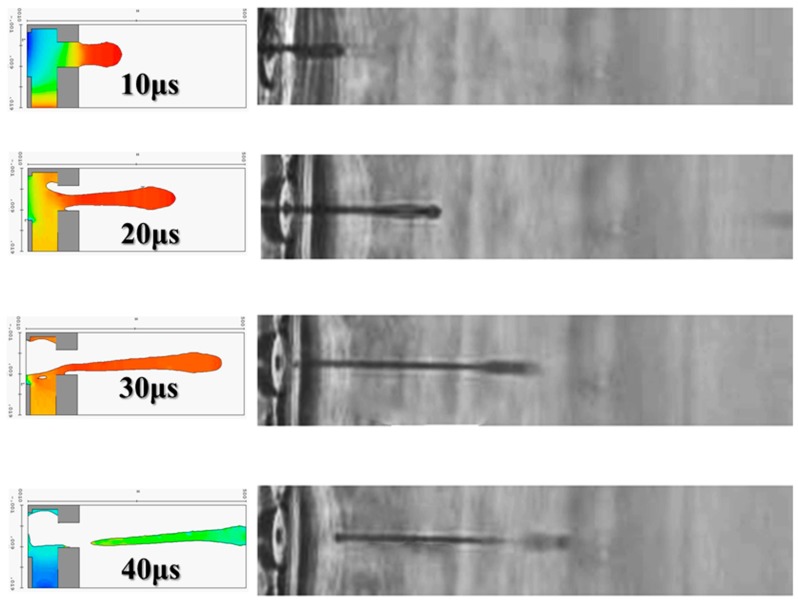
Droplet injection scenario and observation.

The objective of this research project is to increase the flow rate of injection, which requires a method for increasing the frequency of injection. When the inkjet frequency was increased from 18 to 26 kHz, and the ejection frequency was raised to 26 kHz, the traditional single-channel injection cavity design was improved. Figure 16 illustrates the operating frequency that was evaluated at 26 kHz, displaying a 3D side view of 10, 20, 30, and 40 μs injection scenarios. For the 26 kHz ejection frequency, this corresponds to an injection cycle of 40 μs. Drops could be observed in the inkjet wafer, validating each of the actual droplet trajectories. Therefore, 110, 120, 130, and 140 μs were used for the second injection stage. The second injection droplet shape and tail length varied from the first injection results, because the fluid entirely filled the injection cavity and had not yet stabilized the flow field. This result implies that the beginning of a second injection event affects the next injection. Each subsequent injection is more severely affected. Similar results were also derived for representative spray cycles of 100 and 200 μs. No satellite droplets with dragging tails were formed in the calculation of the intrinsic region at 100 μs, and there were satellite droplets at the top of the calculation region at 200 μs that were about to leave the area.

The travel time for the whole liquid droplets to be molded into ink ones to spray on paper is 40 μs for one heater. Using this new drive mode, the inkjet can jet the liquid droplets at a speed higher than 26 kHz. The ability to select different power outputs is the latest method in inkjet machines. In other words, we can use the CMOS multiplexer jets matrix addressing method to give an inkjet machine the features of rapid heating, large expansion, and high compression to expel liquid droplets onto printing paper to form dots.

## 5. Conclusions

A smart bubble-jet printhead with a long lifespan was proposed and demonstrated. The multiplexer printhead integrated inkjet nozzle arrays through both standard complementary MOS (CMOS) processes and micromachining technology. The integration of CMOS and enhancement-mode devices, power switches, and a TIJ heater transducer allowed for logic functions to be performed on-chip. This capability was used in the design to address individual jets with even fewer input lines than in matrix addressing. Only 11 input lines were required to address a large number of jets (896 or more).
